# A case of neonatal infection caused by *Streptococcus agalactiae* sequence type 283 in China’s mainland

**DOI:** 10.1093/jacamr/dlag002

**Published:** 2026-01-30

**Authors:** Jiaming Zhang, Huiqiang Fu, Beibei Miao, Jingyi Zhang, Haijian Zhou, Dongke Chen, Biao Kan, Juan Li

**Affiliations:** National Key Laboratory of Intelligent Tracing and Forecasting for Infectious Diseases, National Institute for Communicable Disease Control and Prevention, Chinese Center for Disease Control and Prevention, Beijing, China; National Key Laboratory of Intelligent Tracing and Forecasting for Infectious Diseases, National Institute for Communicable Disease Control and Prevention, Chinese Center for Disease Control and Prevention, Beijing, China; National Key Laboratory of Intelligent Tracing and Forecasting for Infectious Diseases, National Institute for Communicable Disease Control and Prevention, Chinese Center for Disease Control and Prevention, Beijing, China; National Key Laboratory of Intelligent Tracing and Forecasting for Infectious Diseases, National Institute for Communicable Disease Control and Prevention, Chinese Center for Disease Control and Prevention, Beijing, China; National Key Laboratory of Intelligent Tracing and Forecasting for Infectious Diseases, National Institute for Communicable Disease Control and Prevention, Chinese Center for Disease Control and Prevention, Beijing, China; Central & Clinical Laboratory of Sanya People's Hospital, Sanya, Hainan, China; National Key Laboratory of Intelligent Tracing and Forecasting for Infectious Diseases, National Institute for Communicable Disease Control and Prevention, Chinese Center for Disease Control and Prevention, Beijing, China; National Key Laboratory of Intelligent Tracing and Forecasting for Infectious Diseases, National Institute for Communicable Disease Control and Prevention, Chinese Center for Disease Control and Prevention, Beijing, China

## Introduction

*Streptococcus agalactiae*, also known as Group B Streptococcus (GBS), is a leading cause of neonatal sepsis, meningitis and pneumonia worldwide. Sequence type 283 (ST283), a hypervirulent lineage of GBS serotype III, has been implicated in large-scale foodborne outbreaks in Southeast Asia, particularly in Singapore, Hong Kong and Thailand.^1,2^ These outbreaks were primarily linked to consumption of or contact with raw freshwater fish, with >380 reported cases to date (Table S1, available as Supplementary data at *JAC-AMR* Online). While ST283 has been isolated from both humans and animals, its role in neonatal infections has been rarely documented, with only one previous case of early-onset disease (EOD) reported in Hong Kong and a total of four neonatal infections cases reported worldwide.^3,4^ No cases of ST283 infection have been reported in China’s mainland.

Here, we describe the first case of EOD caused by ST283 in China’s mainland, providing evidence for maternal–neonate transmission and highlighting the potential for this clone to colonize mothers and infect neonates.

## The study

The index case was a term neonate (38 + 3 weeks gestation, birth weight 3220 g) delivered vaginally to a healthy 26-year-old mother with no history of fever or infectious diseases. The neonate developed respiratory distress and lethargy within 6 hours of birth. Blood culture of the neonate grew *S. agalactiae* and the isolate was named as ZJSX030. The neonate achieved complete clinical recovery following a 10-day course of intravenous penicillin therapy.

We performed third-generation sequencing (PacBio) on the isolate, followed by *de novo* assembly using Unicycler (v.0.5.0) to generate its complete genomic map. The sequence data were deposited in the National Genomics Data Center under BioProject ID NMDC10020060. In silico MLST confirmed the isolate as ST283. To compare this isolate with other ST283 isolates, we downloaded all available *S. agalactiae* ST283 genomic sequences from the PubMLST (https://pubmlst.org/). Detailed information on these 241 isolates was presented in Table S2. A phylogenetic tree for all genomes from the PubMLST database and ZJSX030 was constructed based on their core-genome SNPs using Snippy version 4.4.6 and FastTree. The genome of isolate SG-M158 (GCA_002197265.1) was used as a reference, which represents a fully closed, high-quality genome of the ST283 lineage.^5^

**Figure 1.**
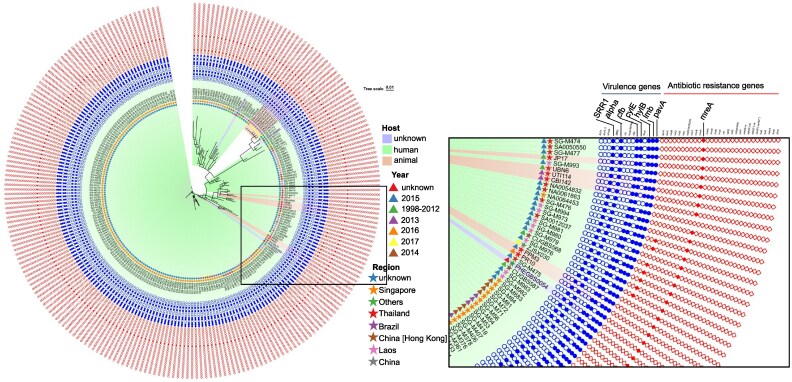
Genetic characterization of the *S. agalactiae* ST283 strain obtained from the blood of a neonate and download data. Phylogenetic tree of the 241 GBS genomes with the reference strains. The *S. agalactiae* SG-M158, which was sequenced using third-generation sequencing, was used as the reference strain. Blue circles denote virulence genes and red diamonds denote antibiotic resistance genes. Empty shapes represent the absence and filled shapes represent the presence of the respective genes. The right plot is a magnified view of the region within the red square.

Phylogenetic analysis showed high genetic homogeneity in ST283 isolates across hosts, geographic regions, and previously reported isolates outside China’s mainland. As shown in Figure 1, the ZJSX030 isolate is closely related to two animal-derived ST283 isolates from Thailand. As previously reported, ST283 is a zoonotic Asian GBS clone capable of colonizing and infecting various farmed freshwater fish, causing unusually severe invasive disease in humans. To elucidate the relationship between ZJSX030 and the two subtypes, we analysed the SNPs between them (Figure S1). There is minimal difference between animal-derived and human-derived ST283 strains, with ∼15 SNPs. Interesting, we found *guaA*, *thiM*, *gtaB*, *rcsC*, *glnA*, *pepO*, *norm*, *rsmA* and *licR* harboured missense mutations. Mutations in *guaA* may enhance the production of nucleotide precursors, which not only accelerates bacterial proliferation within the host but also improves the bacterium’s ability to tolerate nutrient scarcity.^6^  *rcsC* regulates capsule synthesis and stress responses, thereby enhancing immune escape and increasing the bacterium’s adaptability to the host environment.^7^ The SNPs in these genes may confer an adaptive advantage in human, necessitating further research.

To initially assess the virulence and resistance potential of this strain, we performed a comprehensive analysis of its virulence factors and antimicrobial resistance patterns (Figure 1). ZJSX030 carried the characteristic ST283 virulence gene suite, including: *cfb*, *cyl*E, *pav*A, α protein family (*Alpha*), *Srr*1, *hyl*B and *Lmb*. Genome screening identified the tetracycline resistance gene *tet*M and the macrolide resistance gene *mef*A in strain ZJSX030. These results were consistent with the antimicrobial susceptibility test findings, which showed resistance to tetracycline (MIC >8 mg/L) and erythromycin (MIC >4 m/L), as well as susceptibility to penicillin, Clindamycin, levofloxacin, vancomycin, linezolid and ceftriaxone.^8^

## Discussion

This is the first report of ST283-associated neonatal sepsis in China’s mainland. The isolate’s genomic similarity to fish-derived strains from Thailand supports the hypothesis that ST283 is a zoonotic clone. The mother of the neonate denied any exposure to fish and aquatic products within 1 month before delivery. Although we were unable to obtain vaginal or rectal swabs from the mother due to the retrospective nature of the case, the timing of symptom onset (within 24 hours of birth) strongly supports EOD resulting from vertical transmission.^9^ This case expands the epidemiologic profile of ST283 beyond foodborne illness and highlights its potential as a neonatal pathogen.^4^

### Conclusions

*S. agalactiae* ST283 is no longer restricted to foodborne transmission. Its emergence as a cause of neonatal sepsis in China’s mainland underscores the need for enhanced antenatal surveillance, molecular epidemiology and public health preparedness to prevent further spread of this hypervirulent clone. We recommend investigating gastrointestinal colonization as a potential reservoir for ST283.

## Supplementary Material

dlag002_Supplementary_Data

## Supplementary data

Figure S1 and Tables S1 and S2 are available as Supplementary data at *JAC-AMR* Online.

## References

[1] Aiewsakun P, Ruangchai W, Thawornwattana Y et al Genomic epidemiology of *Streptococcus agalactiae* ST283 in Southeast Asia. Sci Rep 2022; 12: 4185. 10.1038/s41598-022-08097-0 35264716 PMC8907273

[2] Chau ML, Chen SL, Yap M et al Group B Streptococcus infections caused by improper sourcing and handling of fish for raw consumption, Singapore, 2015–2016. Emerg Infect Dis 2017; 23: 2002–10. 10.3201/eid2312.170596 29148967 PMC5708258

[3] Boonsilp S, Nealiga MJ, Wangchuk K et al Differential interaction between invasive Thai group B *Streptococcus Sequence* type 283 and caco-2 cells. Microorganisms 2022; 10: 1917. 10.3390/microorganisms10101917 36296194 PMC9611625

[4] Ip M, Ang I, Fung K et al Hypervirulent clone of group B *Streptococcus Serotype* III sequence type 283, Hong Kong, 1993–2012. Emerg Infect Dis 2016; 22: 1800–3. 10.3201/eid2210.151436 27648702 PMC5038432

[5] Kalimuddin S, Chen SL, Lim CTK et al 2015 epidemic of severe *Streptococcus agalactiae* sequence type 283 infections in Singapore associated with the consumption of raw freshwater fish: a detailed analysis of clinical, epidemiological, and bacterial sequencing data. Clin Infect Dis 2017; 64: S145–52. 10.1093/cid/cix021 28475781

[6] Ipe DS, Sullivan MJ, Goh KGK et al Conserved bacterial *de novo* guanine biosynthesis pathway enables microbial survival and colonization in the environmental niche of the urinary tract. ISME J 2021; 15: 2158–62. 10.1038/s41396-021-00934-w 33649549 PMC8245529

[7] Kessler NG, Caraballo Delgado DM, Shah NK et al Exopolysaccharide anchoring creates an extreme resistance to sedimentation. J Bacteriol 2021; 203: e00023-21. 10.1128/JB.00023-21 33753470 PMC8117528

[8] Muthanna A, Desa MNM, Alsalemi W et al Phenotypic and genotypic comparison of pathogenic group B *Streptococcus* isolated from human and cultured tilapia (*Oreochromis* species) in Malaysia. Comp Immunol Microbiol Infect Dis 2023; 97: 101993. 10.1016/j.cimid.2023.101993 37167694

[9] Barkham T, Tang WY, Wang YC et al Human fecal carriage of *Streptococcus agalactiae* sequence type 283, Thailand. Emerg Infect Dis 2023; 29: 1627–9. 10.3201/eid2908.230098 37486205 PMC10370859

