# Supplementary material for: A case of neonatal infection caused by Streptococcus agalactiae sequence type 283 in China’s mainland
Source: JAC Antimicrob Resist. 2026 Jan 30;8(1):dlag002. doi: 10.1093/jacamr/dlag002 (PMC12856657; doi:10.1093/jacamr/dlag002)
Supplement: dlag002_Supplementary_Data [file dlag002_supplementary_data.docx]

**Figure S1 Analysis of SNP between PPM3, PR10, and ZJSX030. SNPs are highlighted in red, and >90% are missense variants. The gene**

**names and genomic location are displayed for each SNP site.**

**Table S1. Patients with group B Streptococcus (GBS) ST 283 reported up to 2024.**

| Date | Countries/regions | Patients | Source |
| --- | --- | --- | --- |
| 1995-2012 | Hong Kong | 3 neonates (one infant had early-onset sepsis, and 2 had lateonset  Sepsis), 1 pregnant women, and 44 nonpregnant adults | PMC5038432 |
| 2012-2016 | Thailand | 1 neonates, and 31 adults | PMC9611625 |
| 2012-2016, 2018 | Thailand | 12 adults | PMC8907273 |
| 2015 | Singapore | 146 adults | PMID: 28475781 |
| 2018.11-2019.07 | Thailand | 210 adults | PMC11920070 |
| 2018.11 | Laos | 2 adults | PMC9607938 |
| 2019.07-2020.12 | Malaysia | 2 adults | PMID: 37167694 |
| 2021.02-2023.01 | Iran | 2 adults | PMC11438095 |
| 2021.09-11 | Hong Kong | 95 adults | PMC11682793 |
| 2024.08-09 | Hong Kong | 131 adults | PMC12247001 |

Table S2 The information of strains used in this study.

| **Sample name** | **Country/ Region** | **Year** | **Isolation Source** | **pili-related genes** | **virulence genes** | | | | | | | | | | **drug resistant gene** | | |
| --- | --- | --- | --- | --- | --- | --- | --- | --- | --- | --- | --- | --- | --- | --- | --- | --- | --- |
|  |  |  |  | PILUS | HVGA | SRR1 | SRR2 | alpha | rib | cfb | cylE | hylB | lmb | pavA | tetM | ermT | mreA |
| STIR-CD-25 | Unknown | Unknown | Unknown | 1+2a | 0 | 1 | 0 | 1 | 0 | 1 | 1 | 1 | 1 | 1 | 1 | 0 | 1 |
| SG-M1 | Singapore | 2015 | human | 1+2a | 0 | 1 | 0 | 1 | 0 | 1 | 1 | 1 | 1 | 1 | 0 | 0 | 1 |
| ERR1659855 | The Netherlands | Unknown | Unknown | 1+2a | 0 | 1 | 0 | 1 | 0 | 1 | 1 | 1 | 0 | 1 | 1 | 0 | 1 |
| JP17 | Thailand | 2012 | other animal | 1+2a | 0 | 1 | 0 | 1 | 0 | 1 | 1 | 1 | 1 | 1 | 0 | 0 | 1 |
| CBI142 | Thailand | 2013 | other animal | 1+2a | 0 | 1 | 0 | 1 | 0 | 1 | 1 | 1 | 1 | 1 | 0 | 0 | 1 |
| UTI114 | Thailand | 2013 | other animal | 1+2a | 0 | 1 | 0 | 1 | 0 | 1 | 1 | 1 | 1 | 1 | 0 | 0 | 1 |
| PPM3 | Thailand | 2015 | other animal | 1+2a | 0 | 1 | 0 | 1 | 0 | 1 | 1 | 1 | 1 | 1 | 0 | 0 | 1 |
| PR10 | Thailand | 2016 | other animal | 1+2a | 0 | 1 | 0 | 1 | 0 | 1 | 1 | 1 | 1 | 1 | 0 | 0 | 1 |
| UBN6 | Thailand | 2012 | other animal | 1+2a | 0 | 1 | 0 | 1 | 0 | 1 | 1 | 1 | 1 | 1 | 0 | 0 | 1 |
| SA01AQUAVET | Brazil | 2017 | other animal | 1+2a | 0 | 1 | 0 | 1 | 0 | 1 | 1 | 1 | 0 | 1 | 1 | 0 | 1 |
| SA06AQUAVET | Brazil | 2017 | other animal | 1+2a | 0 | 1 | 0 | 1 | 0 | 1 | 1 | 1 | 0 | 1 | 1 | 0 | 1 |
| SA12AQUAVET | Brazil | 2017 | other animal | 1+2a | 0 | 0 | 0 | 1 | 0 | 1 | 1 | 1 | 0 | 1 | 1 | 0 | 1 |
| SA22AQUAVET | Brazil | 2017 | other animal | 1+2a | 0 | 1 | 0 | 1 | 0 | 1 | 1 | 1 | 0 | 1 | 1 | 0 | 1 |
| SA90AQUAVET | Brazil | 2017 | other animal | 1+2a | 0 | 1 | 0 | 1 | 0 | 1 | 1 | 1 | 0 | 1 | 1 | 0 | 1 |
| SA98AQUAVET | Brazil | 2017 | other animal | 1+2a | 0 | 1 | 0 | 1 | 0 | 1 | 1 | 1 | 0 | 1 | 1 | 0 | 1 |
| SApx2AQUAVET | Brazil | 2017 | other animal | 1+2a | 0 | 1 | 0 | 1 | 0 | 1 | 1 | 1 | 0 | 1 | 1 | 0 | 1 |
| SApx7AQUAVET | Brazil | 2016 | other animal | 1+2a | 0 | 1 | 0 | 1 | 0 | 1 | 1 | 1 | 0 | 1 | 1 | 0 | 1 |
| SG-M121 | Singapore | 2015 | human | 1+2a | 0 | 1 | 0 | 1 | 0 | 1 | 1 | 1 | 1 | 1 | 0 | 0 | 1 |
| SG-M151 | Singapore | 2015 | human | 1+2a | 0 | 1 | 0 | 1 | 0 | 1 | 1 | 1 | 1 | 1 | 0 | 0 | 1 |
| SG-M123 | Singapore | 2015 | human | 1+2a | 0 | 1 | 0 | 1 | 0 | 1 | 1 | 1 | 1 | 1 | 0 | 0 | 1 |
| SG-M306 | Singapore | 2015 | human | 1+2a | 0 | 1 | 0 | 1 | 0 | 1 | 1 | 1 | 1 | 1 | 0 | 0 | 1 |
| SG-M309 | Singapore | 2015 | other human | 1+2a | 0 | 1 | 0 | 1 | 0 | 1 | 1 | 1 | 1 | 1 | 0 | 0 | 1 |
| SG-M312 | Singapore | 2015 | human | 1+2a | 0 | 1 | 0 | 1 | 0 | 1 | 1 | 0 | 1 | 1 | 0 | 0 | 1 |
| SG-M32 | Singapore | 2015 | human | 1+2a | 0 | 1 | 0 | 1 | 0 | 1 | 1 | 1 | 1 | 1 | 0 | 0 | 1 |
| SG-M33 | Singapore | 2015 | human | 1+2a | 0 | 1 | 0 | 1 | 0 | 1 | 1 | 1 | 1 | 1 | 0 | 0 | 1 |
| SG-M361 | Singapore | 2013 | human | 1+2a | 0 | 1 | 0 | 1 | 0 | 1 | 1 | 1 | 1 | 1 | 0 | 0 | 1 |
| SG-M376 | Singapore | 2013 | human | 1+2a | 0 | 1 | 0 | 1 | 0 | 1 | 1 | 1 | 1 | 1 | 0 | 0 | 1 |
| SG-M378 | Singapore | 2013 | human | 1+2a | 0 | 1 | 0 | 1 | 0 | 1 | 1 | 1 | 1 | 1 | 0 | 0 | 1 |
| SG-M406 | Singapore | 2014 | human | 1+2a | 0 | 1 | 0 | 1 | 0 | 1 | 1 | 1 | 1 | 1 | 0 | 0 | 1 |
| SG-M153 | Singapore | 2015 | human | 1+2a | 0 | 1 | 0 | 1 | 0 | 1 | 1 | 1 | 1 | 1 | 0 | 0 | 1 |
| SG-M407 | Singapore | 2014 | human | 1+2a | 0 | 1 | 0 | 1 | 0 | 1 | 1 | 1 | 1 | 1 | 0 | 0 | 1 |
| SG-M419 | Singapore | 2014 | human | 1+2a | 0 | 1 | 0 | 1 | 0 | 1 | 1 | 1 | 1 | 1 | 0 | 0 | 1 |
| SG-M424 | Singapore | 2015 | human | 1+2a | 0 | 1 | 0 | 1 | 0 | 1 | 1 | 1 | 1 | 1 | 0 | 0 | 1 |
| SG-M430 | Singapore | 2015 | human | 1+2a | 0 | 1 | 0 | 1 | 0 | 1 | 1 | 1 | 1 | 1 | 0 | 0 | 1 |
| SG-M431 | Singapore | 2015 | human | 1+2a | 0 | 1 | 0 | 1 | 0 | 1 | 1 | 1 | 1 | 1 | 0 | 0 | 1 |
| SG-M432 | Singapore | 2015 | human | 1+2a | 0 | 1 | 0 | 1 | 0 | 1 | 1 | 1 | 1 | 1 | 0 | 0 | 1 |
| SG-M434 | Singapore | 2015 | human | 1+2a | 0 | 1 | 0 | 1 | 0 | 1 | 1 | 1 | 1 | 1 | 0 | 0 | 1 |
| SG-M155 | Singapore | 2015 | human | 1+2a | 0 | 1 | 0 | 1 | 0 | 1 | 1 | 1 | 1 | 1 | 0 | 0 | 1 |
| SG-M435 | Singapore | 2015 | human | 1+2a | 0 | 1 | 0 | 1 | 0 | 1 | 1 | 1 | 1 | 1 | 0 | 0 | 1 |
| SG-M442 | Singapore | 2015 | human | 1+2a | 0 | 1 | 0 | 1 | 0 | 1 | 1 | 1 | 1 | 1 | 0 | 0 | 1 |
| SG-M439 | Singapore | 2015 | human | 1+2a | 0 | 1 | 0 | 1 | 0 | 1 | 1 | 1 | 1 | 1 | 0 | 0 | 1 |
| SG-M443 | Singapore | 2015 | human | 1+2a | 0 | 1 | 0 | 1 | 0 | 1 | 1 | 1 | 1 | 1 | 0 | 0 | 1 |
| SG-M444 | Singapore | 2015 | human | 1+2a | 0 | 1 | 0 | 1 | 0 | 1 | 1 | 1 | 1 | 1 | 0 | 0 | 1 |
| SG-M449 | Singapore | 2015 | human | 1+2a | 0 | 1 | 0 | 1 | 0 | 1 | 1 | 1 | 1 | 1 | 0 | 0 | 1 |
| SG-M448 | Singapore | 2015 | human | 1+2a | 0 | 1 | 0 | 1 | 0 | 1 | 1 | 1 | 1 | 1 | 0 | 0 | 1 |
| SG-M45 | Singapore | 2013 | human | 1+2a | 0 | 1 | 0 | 1 | 0 | 1 | 1 | 1 | 1 | 1 | 0 | 0 | 1 |
| SG-M450 | Singapore | 2015 | human | 1+2a | 0 | 1 | 0 | 1 | 0 | 1 | 1 | 1 | 1 | 1 | 0 | 0 | 1 |
| SG-M451 | Singapore | 2015 | human | 1+2a | 0 | 1 | 0 | 1 | 0 | 1 | 1 | 1 | 1 | 1 | 0 | 0 | 1 |
| SG-M156 | Singapore | 2015 | human | 1+2a | 0 | 1 | 0 | 1 | 0 | 1 | 1 | 1 | 1 | 1 | 0 | 0 | 1 |
| SG-M452 | Singapore | 2015 | human | 1+2a | 0 | 1 | 0 | 1 | 0 | 1 | 1 | 1 | 1 | 1 | 0 | 0 | 1 |
| SG-M456 | Singapore | 2015 | human | 1+2a | 0 | 1 | 0 | 1 | 0 | 1 | 1 | 1 | 1 | 1 | 0 | 0 | 1 |
| SG-M457 | Singapore | 2015 | human | 1+2a | 0 | 1 | 0 | 1 | 0 | 1 | 1 | 1 | 1 | 1 | 0 | 0 | 1 |
| SG-M474 | Thailand | 2015 | human | 1+2a | 0 | 1 | 0 | 1 | 0 | 1 | 1 | 1 | 1 | 1 | 0 | 0 | 1 |
| SG-M475 | Thailand | 2015 | human | 1+2a | 0 | 1 | 0 | 1 | 0 | 1 | 1 | 1 | 1 | 1 | 0 | 0 | 1 |
| SG-M476 | Thailand | 2015 | human | 1+2a | 0 | 1 | 0 | 1 | 0 | 1 | 1 | 1 | 1 | 1 | 0 | 0 | 1 |
| SG-M477 | Thailand | 2015 | human | 1+2a | 0 | 1 | 0 | 1 | 0 | 1 | 1 | 1 | 1 | 1 | 0 | 0 | 1 |
| SG-M50 | Singapore | 2013 | human | 1+2a | 0 | 1 | 0 | 1 | 0 | 1 | 1 | 1 | 1 | 1 | 0 | 0 | 1 |
| SG-M53 | Singapore | 2013 | human | 1+2a | 0 | 1 | 0 | 1 | 0 | 1 | 1 | 1 | 1 | 1 | 0 | 0 | 1 |
| SG-M157 | Singapore | 2015 | human | 1+2a | 0 | 1 | 0 | 1 | 0 | 1 | 1 | 1 | 1 | 1 | 0 | 0 | 1 |
| SG-M54 | Singapore | 2013 | human | 1+2a | 0 | 1 | 0 | 1 | 0 | 1 | 1 | 1 | 1 | 1 | 0 | 0 | 1 |
| SG-M56 | Singapore | 2013 | human | 1+2a | 0 | 1 | 0 | 1 | 0 | 1 | 1 | 1 | 1 | 1 | 0 | 0 | 1 |
| SG-M62 | Singapore | 2013 | human | 1+2a | 0 | 1 | 0 | 1 | 0 | 1 | 1 | 1 | 1 | 1 | 0 | 0 | 1 |
| SG-M64 | Singapore | 2013 | human | 1+2a | 0 | 1 | 0 | 1 | 0 | 1 | 1 | 1 | 1 | 1 | 0 | 0 | 1 |
| SG-M71 | Singapore | 2014 | human | 1+2a | 0 | 1 | 0 | 1 | 0 | 1 | 1 | 1 | 1 | 1 | 0 | 0 | 1 |
| SG-M72 | Singapore | 2014 | human | 1+2a | 0 | 1 | 0 | 1 | 0 | 1 | 1 | 1 | 1 | 1 | 0 | 0 | 1 |
| SG-M74 | Singapore | 2014 | human | 1+2a | 0 | 1 | 0 | 1 | 0 | 1 | 1 | 1 | 1 | 1 | 0 | 0 | 1 |
| SG-M78 | Singapore | 2014 | human | 1+2a | 0 | 1 | 0 | 1 | 0 | 1 | 1 | 1 | 1 | 1 | 0 | 0 | 1 |
| SG-M80 | Singapore | 2014 | human | 1+2a | 0 | 1 | 0 | 1 | 0 | 1 | 1 | 1 | 1 | 1 | 0 | 0 | 1 |
| SG-M81 | Singapore | 2014 | human | 1+2a | 0 | 1 | 0 | 1 | 0 | 1 | 1 | 1 | 1 | 1 | 0 | 0 | 1 |
| SG-M158 | Singapore | 2015 | human | 1+2a | 0 | 1 | 0 | 1 | 0 | 1 | 1 | 1 | 1 | 1 | 0 | 0 | 1 |
| SG-M82 | Singapore | 2014 | human | 1+2a | 0 | 1 | 0 | 1 | 0 | 1 | 1 | 1 | 1 | 1 | 0 | 0 | 1 |
| SG-M84 | Singapore | 2014 | human | 1+2a | 0 | 1 | 0 | 1 | 0 | 1 | 1 | 1 | 1 | 1 | 0 | 0 | 1 |
| SG-M90 | Singapore | 2014 | human | 1+2a | 0 | 1 | 0 | 1 | 0 | 1 | 1 | 1 | 1 | 1 | 0 | 0 | 1 |
| SG-M159 | Singapore | 2015 | human | 1+2a | 0 | 1 | 0 | 1 | 0 | 1 | 1 | 1 | 1 | 1 | 0 | 0 | 1 |
| SG-M162 | Singapore | 2015 | human | 1+2a | 0 | 1 | 0 | 1 | 0 | 1 | 1 | 1 | 1 | 1 | 0 | 0 | 1 |
| SG-M163 | Singapore | 2015 | human | 1+2a | 0 | 1 | 0 | 1 | 0 | 1 | 1 | 1 | 1 | 1 | 0 | 0 | 1 |
| SG-M165 | Singapore | 2015 | human | 1+2a | 0 | 1 | 0 | 1 | 0 | 1 | 1 | 1 | 1 | 1 | 0 | 0 | 1 |
| SG-M166 | Singapore | 2015 | human | 1+2a | 0 | 1 | 0 | 1 | 0 | 1 | 1 | 1 | 1 | 1 | 0 | 0 | 1 |
| SG-M168 | Singapore | 2015 | human | 1+2a | 0 | 1 | 0 | 1 | 0 | 1 | 1 | 1 | 1 | 1 | 0 | 0 | 1 |
| SG-M126 | Singapore | 2015 | human | 1+2a | 0 | 1 | 0 | 1 | 0 | 1 | 1 | 1 | 1 | 1 | 0 | 0 | 1 |
| SG-M169 | Singapore | 2015 | human | 1+2a | 0 | 1 | 0 | 1 | 0 | 1 | 1 | 1 | 1 | 1 | 0 | 0 | 1 |
| SG-M170 | Singapore | 2015 | human | 1+2a | 0 | 1 | 0 | 1 | 0 | 1 | 1 | 1 | 1 | 1 | 0 | 0 | 1 |
| SG-M172 | Singapore | 2015 | human | 1+2a | 0 | 1 | 0 | 1 | 0 | 1 | 1 | 1 | 1 | 1 | 0 | 0 | 1 |
| SG-M173 | Singapore | 2015 | human | 1+2a | 0 | 1 | 0 | 1 | 0 | 1 | 1 | 1 | 1 | 1 | 0 | 0 | 1 |
| SG-M175 | Singapore | 2015 | human | 1+2a | 0 | 1 | 0 | 1 | 0 | 1 | 1 | 1 | 1 | 1 | 0 | 0 | 1 |
| SG-M177 | Singapore | 2015 | human | 1+2a | 0 | 1 | 0 | 1 | 0 | 1 | 1 | 1 | 1 | 1 | 0 | 0 | 1 |
| SG-M178 | Singapore | 2015 | human | 1+2a | 0 | 1 | 0 | 1 | 0 | 1 | 1 | 1 | 1 | 1 | 0 | 0 | 1 |
| SG-M181 | Singapore | 2015 | human | 1+2a | 0 | 1 | 0 | 1 | 0 | 1 | 1 | 1 | 1 | 1 | 0 | 0 | 1 |
| SG-M127 | Singapore | 2015 | human | 1+2a | 0 | 1 | 0 | 1 | 0 | 1 | 1 | 1 | 1 | 1 | 0 | 0 | 1 |
| SG-M183 | Singapore | 2015 | human | 1+2a | 0 | 1 | 0 | 1 | 0 | 1 | 1 | 1 | 1 | 1 | 0 | 0 | 1 |
| SG-M191 | Singapore | 2015 | human | 1+2a | 0 | 1 | 0 | 1 | 0 | 1 | 1 | 1 | 1 | 1 | 0 | 0 | 1 |
| SG-M192 | Singapore | 2015 | human | 1+2a | 0 | 1 | 0 | 1 | 0 | 1 | 1 | 1 | 1 | 1 | 0 | 0 | 1 |
| SG-M193 | Singapore | 2015 | human | 1+2a | 0 | 1 | 0 | 1 | 0 | 1 | 1 | 1 | 1 | 1 | 0 | 0 | 1 |
| SG-M194 | Singapore | 2015 | human | 1+2a | 0 | 1 | 0 | 1 | 0 | 1 | 1 | 1 | 1 | 1 | 0 | 0 | 1 |
| SG-M195 | Singapore | 2015 | human | 1+2a | 0 | 1 | 0 | 1 | 0 | 1 | 1 | 1 | 1 | 1 | 0 | 0 | 1 |
| SG-M196 | Singapore | 2015 | human | 1+2a | 0 | 1 | 0 | 1 | 0 | 1 | 1 | 1 | 1 | 1 | 0 | 0 | 1 |
| SG-M197 | Singapore | 2015 | human | 1+2a | 0 | 1 | 0 | 1 | 0 | 1 | 1 | 1 | 1 | 1 | 0 | 0 | 1 |
| SG-M199 | Singapore | 2015 | human | 1+2a | 0 | 1 | 0 | 1 | 0 | 1 | 1 | 1 | 1 | 1 | 0 | 0 | 1 |
| SG-M200 | Singapore | 2015 | human | 1+2a | 0 | 1 | 0 | 1 | 0 | 1 | 1 | 1 | 1 | 1 | 0 | 0 | 1 |
| SG-M137 | Singapore | 2015 | human | 1+2a | 0 | 1 | 0 | 1 | 0 | 1 | 1 | 1 | 1 | 1 | 0 | 0 | 1 |
| SG-M202 | Singapore | 2015 | human | 1+2a | 0 | 1 | 0 | 1 | 0 | 1 | 1 | 1 | 1 | 1 | 0 | 0 | 1 |
| SG-M203 | Singapore | 2015 | human | 1+2a | 0 | 1 | 0 | 1 | 0 | 1 | 1 | 1 | 1 | 1 | 0 | 0 | 1 |
| SG-M205 | Singapore | 2015 | human | 1+2a | 0 | 1 | 0 | 1 | 0 | 1 | 1 | 1 | 1 | 1 | 0 | 0 | 1 |
| SG-M207 | Singapore | 2015 | human | 1+2a | 0 | 1 | 0 | 1 | 0 | 1 | 1 | 1 | 1 | 1 | 0 | 0 | 1 |
| SG-M210 | Singapore | 2015 | human | 1+2a | 0 | 1 | 0 | 1 | 0 | 1 | 1 | 1 | 1 | 1 | 0 | 0 | 1 |
| SG-M209 | Singapore | 2015 | human | 1+2a | 0 | 1 | 0 | 1 | 0 | 1 | 1 | 1 | 1 | 1 | 0 | 0 | 1 |
| SG-M213 | Singapore | 2015 | human | 1+2a | 0 | 1 | 0 | 1 | 0 | 1 | 1 | 1 | 1 | 1 | 0 | 0 | 1 |
| SG-M214 | Singapore | 2015 | human | 1+2a | 0 | 1 | 0 | 1 | 0 | 1 | 1 | 1 | 1 | 1 | 0 | 0 | 1 |
| SG-M216 | Singapore | 2015 | human | 1+2a | 0 | 1 | 0 | 1 | 0 | 1 | 1 | 1 | 1 | 1 | 0 | 0 | 1 |
| SG-M217 | Singapore | 2015 | human | 1+2a | 0 | 1 | 0 | 1 | 0 | 1 | 1 | 1 | 1 | 1 | 0 | 0 | 1 |
| SG-M142 | Singapore | 2015 | human | 1+2a | 0 | 1 | 0 | 1 | 0 | 1 | 1 | 1 | 1 | 1 | 0 | 0 | 1 |
| SG-M218 | Singapore | 2015 | human | 1+2a | 0 | 1 | 0 | 1 | 0 | 1 | 1 | 1 | 1 | 1 | 0 | 0 | 1 |
| SG-M219 | Singapore | 2015 | human | 1+2a | 0 | 1 | 0 | 1 | 0 | 1 | 1 | 1 | 1 | 1 | 0 | 0 | 1 |
| SG-M220 | Singapore | 2015 | human | 1+2a | 0 | 1 | 0 | 1 | 0 | 1 | 1 | 1 | 1 | 1 | 0 | 0 | 1 |
| SG-M222 | Singapore | 2015 | human | 1+2a | 0 | 1 | 0 | 1 | 0 | 1 | 1 | 1 | 1 | 1 | 0 | 0 | 1 |
| SG-M223 | Singapore | 2015 | human | 1+2a | 0 | 1 | 0 | 1 | 0 | 1 | 1 | 1 | 1 | 1 | 0 | 0 | 1 |
| SG-M224 | Singapore | 2015 | human | 1+2a | 0 | 1 | 0 | 1 | 0 | 1 | 1 | 1 | 1 | 1 | 0 | 0 | 1 |
| SG-M226 | Singapore | 2015 | human | 1+2a | 0 | 1 | 0 | 1 | 0 | 1 | 1 | 1 | 1 | 1 | 0 | 0 | 1 |
| SG-M227 | Singapore | 2015 | human | 1+2a | 0 | 1 | 0 | 1 | 0 | 1 | 1 | 1 | 1 | 1 | 0 | 0 | 1 |
| SG-M228 | Singapore | 2015 | human | 1+2a | 0 | 1 | 0 | 1 | 0 | 1 | 1 | 1 | 1 | 1 | 0 | 0 | 1 |
| SG-M229 | Singapore | 2015 | human | 1+2a | 0 | 1 | 0 | 1 | 0 | 1 | 1 | 1 | 1 | 1 | 0 | 0 | 1 |
| SG-M143 | Singapore | 2015 | human | 1+2a | 0 | 1 | 0 | 1 | 0 | 1 | 1 | 1 | 1 | 1 | 0 | 0 | 1 |
| SG-M230 | Singapore | 2015 | human | 1+2a | 0 | 1 | 0 | 1 | 0 | 1 | 1 | 1 | 1 | 1 | 0 | 0 | 1 |
| SG-M232 | Singapore | 2015 | human | 1+2a | 0 | 1 | 0 | 1 | 0 | 1 | 1 | 1 | 1 | 1 | 0 | 0 | 1 |
| SG-M234 | Singapore | 2015 | human | 1+2a | 0 | 1 | 0 | 1 | 0 | 1 | 1 | 1 | 1 | 1 | 0 | 0 | 1 |
| SG-M236 | Singapore | 2015 | human | 1+2a | 0 | 1 | 0 | 1 | 0 | 1 | 1 | 1 | 1 | 1 | 0 | 0 | 1 |
| SG-M237 | Singapore | 2015 | human | 1+2a | 0 | 1 | 0 | 1 | 0 | 1 | 1 | 1 | 1 | 1 | 0 | 0 | 1 |
| SG-M238 | Singapore | 2015 | human | 1+2a | 0 | 1 | 0 | 1 | 0 | 1 | 1 | 1 | 1 | 1 | 0 | 0 | 1 |
| SG-M240 | Singapore | 2015 | human | 1+2a | 0 | 1 | 0 | 1 | 0 | 1 | 1 | 1 | 1 | 1 | 0 | 0 | 1 |
| SG-M242 | Singapore | 2015 | human | 1+2a | 0 | 1 | 0 | 1 | 0 | 1 | 1 | 1 | 1 | 1 | 0 | 0 | 1 |
| SG-M243 | Singapore | 2015 | human | 1+2a | 0 | 1 | 0 | 1 | 0 | 1 | 1 | 1 | 1 | 1 | 0 | 0 | 1 |
| SG-M244 | Singapore | 2015 | human | 1+2a | 0 | 1 | 0 | 1 | 0 | 1 | 1 | 1 | 1 | 1 | 0 | 0 | 1 |
| SG-M145 | Singapore | 2015 | human | 1+2a | 0 | 1 | 0 | 1 | 0 | 1 | 1 | 1 | 1 | 1 | 0 | 0 | 1 |
| SG-M248 | Singapore | 2015 | human | 1+2a | 0 | 1 | 0 | 1 | 0 | 1 | 1 | 1 | 1 | 1 | 0 | 0 | 1 |
| SG-M249 | Singapore | 2015 | other human | 1+2a | 0 | 1 | 0 | 1 | 0 | 1 | 1 | 1 | 1 | 1 | 0 | 0 | 1 |
| SG-M250 | Singapore | 2015 | human | 1+2a | 0 | 1 | 0 | 1 | 0 | 1 | 1 | 1 | 1 | 1 | 0 | 0 | 1 |
| SG-M251 | Singapore | 2015 | human | 1+2a | 0 | 1 | 0 | 1 | 0 | 1 | 1 | 1 | 1 | 1 | 0 | 0 | 1 |
| SG-M253 | Singapore | 2015 | human | 1+2a | 0 | 1 | 0 | 1 | 0 | 1 | 1 | 1 | 1 | 1 | 0 | 0 | 1 |
| SG-M256 | Singapore | 2015 | human | 1+2a | 0 | 1 | 0 | 1 | 0 | 1 | 1 | 1 | 1 | 1 | 0 | 0 | 1 |
| SG-M257 | Singapore | 2015 | other human | 1+2a | 0 | 1 | 0 | 1 | 0 | 1 | 1 | 1 | 1 | 1 | 0 | 0 | 1 |
| SG-M261 | Singapore | 2015 | human | 1+2a | 0 | 1 | 0 | 1 | 0 | 1 | 1 | 1 | 1 | 1 | 0 | 0 | 1 |
| SG-M258 | Singapore | 2015 | other human | 1+2a | 0 | 1 | 0 | 1 | 0 | 1 | 1 | 1 | 1 | 1 | 0 | 0 | 1 |
| SG-M274 | Singapore | 2015 | human | 1+2a | 0 | 1 | 0 | 1 | 0 | 1 | 1 | 1 | 1 | 1 | 0 | 0 | 1 |
| SG-M147 | Singapore | 2015 | human | 1+2a | 0 | 1 | 0 | 1 | 0 | 1 | 1 | 1 | 1 | 1 | 0 | 0 | 1 |
| SG-M278 | Singapore | 2015 | human | 1+2a | 0 | 1 | 0 | 1 | 0 | 1 | 1 | 1 | 1 | 1 | 0 | 0 | 1 |
| SG-M279 | Singapore | 2015 | human | 1+2a | 0 | 1 | 0 | 1 | 0 | 1 | 1 | 1 | 1 | 1 | 0 | 0 | 1 |
| SG-M280 | Singapore | 2015 | human | 1+2a | 0 | 1 | 0 | 1 | 0 | 1 | 1 | 1 | 1 | 1 | 0 | 0 | 1 |
| SG-M281 | Singapore | 2015 | human | 1+2a | 0 | 1 | 0 | 1 | 0 | 1 | 1 | 1 | 1 | 1 | 0 | 0 | 1 |
| SG-M282 | Singapore | 2015 | other human | 1+2a | 0 | 1 | 0 | 1 | 0 | 1 | 1 | 1 | 1 | 1 | 0 | 0 | 1 |
| SG-M284 | Singapore | 2015 | human | 1+2a | 0 | 1 | 0 | 1 | 0 | 1 | 1 | 1 | 1 | 1 | 0 | 0 | 1 |
| SG-M287 | Singapore | 2015 | human | 1+2a | 0 | 1 | 0 | 1 | 0 | 1 | 1 | 1 | 1 | 1 | 0 | 0 | 1 |
| SG-M288 | Singapore | 2015 | human | 1+2a | 0 | 1 | 0 | 1 | 0 | 1 | 1 | 1 | 1 | 1 | 0 | 0 | 1 |
| SG-M289 | Singapore | 2015 | human | 1+2a | 0 | 1 | 0 | 1 | 0 | 1 | 1 | 1 | 1 | 1 | 0 | 0 | 1 |
| SG-M29 | Singapore | 2012 | human | 1+2a | 0 | 1 | 0 | 1 | 0 | 1 | 1 | 1 | 1 | 1 | 0 | 0 | 1 |
| SG-M290 | Singapore | 2015 | human | 1+2a | 0 | 1 | 0 | 1 | 0 | 1 | 1 | 1 | 1 | 1 | 0 | 0 | 1 |
| SG-M148 | Singapore | 2015 | human | 1+2a | 0 | 1 | 0 | 1 | 0 | 1 | 1 | 1 | 1 | 1 | 0 | 0 | 1 |
| SG-M291 | Singapore | 2015 | other human | 1+2a | 0 | 1 | 0 | 1 | 0 | 1 | 1 | 1 | 1 | 1 | 0 | 0 | 1 |
| SG-M292 | Singapore | 2015 | other human | 1+2a | 0 | 1 | 0 | 1 | 0 | 1 | 1 | 1 | 1 | 1 | 0 | 0 | 1 |
| SG-M294 | Singapore | 2015 | human | 1+2a | 0 | 1 | 0 | 1 | 0 | 1 | 1 | 1 | 1 | 1 | 0 | 0 | 1 |
| SG-M295 | Singapore | 2015 | human | 1+2a | 0 | 1 | 0 | 1 | 0 | 1 | 1 | 1 | 1 | 1 | 0 | 0 | 1 |
| SG-M297 | Singapore | 2015 | human | 1+2a | 0 | 1 | 0 | 1 | 0 | 1 | 1 | 1 | 1 | 1 | 0 | 0 | 1 |
| SG-M298 | Singapore | 2015 | other human | 1+2a | 0 | 1 | 0 | 1 | 0 | 1 | 1 | 1 | 1 | 1 | 0 | 0 | 1 |
| SG-M299 | Singapore | 2015 | human | 1+2a | 0 | 1 | 0 | 1 | 0 | 1 | 1 | 1 | 1 | 1 | 0 | 0 | 1 |
| SG-M301 | Singapore | 2015 | human | 1+2a | 0 | 1 | 0 | 1 | 0 | 1 | 1 | 1 | 1 | 1 | 0 | 0 | 1 |
| SG-M304 | Singapore | 2015 | human | 1+2a | 0 | 1 | 0 | 1 | 0 | 1 | 1 | 1 | 1 | 1 | 0 | 0 | 1 |
| CUGBS329 | China [Hong Kong] | 2002 | other human | 1+2a | 0 | 1 | 0 | 1 | 0 | 1 | 1 | 1 | 1 | 1 | 1 | 0 | 1 |
| CUGBS522 | China [Hong Kong] | 2005 | human | 1+2a | 0 | 1 | 0 | 1 | 0 | 1 | 1 | 1 | 1 | 1 | 1 | 0 | 1 |
| CUGBS524 | China [Hong Kong] | 2005 | human | 1+2a | 0 | 1 | 0 | 1 | 0 | 1 | 1 | 1 | 1 | 1 | 1 | 0 | 1 |
| CUGBS550 | China [Hong Kong] | 2006 | human | 1+2a | 0 | 1 | 0 | 1 | 0 | 1 | 1 | 1 | 0 | 1 | 1 | 0 | 1 |
| CUGBS551 | China [Hong Kong] | 2006 | human | 1+2a | 0 | 1 | 0 | 1 | 0 | 1 | 1 | 1 | 0 | 1 | 1 | 0 | 1 |
| CUGBS568 | China [Hong Kong] | 2007 | human | 1+2a | 0 | 1 | 0 | 1 | 0 | 1 | 1 | 1 | 1 | 1 | 1 | 0 | 1 |
| CUGBS58 | China [Hong Kong] | 1998 | human | 1+2a | 0 | 1 | 0 | 1 | 0 | 1 | 1 | 1 | 1 | 1 | 1 | 0 | 1 |
| CUGBS581 | China [Hong Kong] | 2007 | human | 1+2a | 0 | 1 | 0 | 1 | 0 | 1 | 1 | 1 | 1 | 1 | 1 | 0 | 1 |
| CUGBS587 | China [Hong Kong] | 2008 | other human | 1+2a | 0 | 1 | 0 | 1 | 0 | 1 | 1 | 1 | 1 | 1 | 0 | 0 | 1 |
| SG-M993 | Laos | 2015 | human | 1+2a | 0 | 1 | 0 | 1 | 0 | 1 | 1 | 1 | 1 | 1 | 0 | 0 | 1 |
| SG-M992 | Laos | 2015 | human | 1+2a | 0 | 1 | 0 | 1 | 0 | 1 | 1 | 1 | 1 | 1 | 0 | 0 | 1 |
| SG-M982 | Laos | 2015 | human | 1+2a | 0 | 1 | 0 | 1 | 0 | 1 | 1 | 1 | 1 | 1 | 0 | 0 | 1 |
| SG-M981 | Laos | 2015 | human | 1+2a | 0 | 1 | 0 | 1 | 0 | 1 | 1 | 1 | 1 | 1 | 1 | 0 | 1 |
| SG-M984 | Laos | 2015 | human | 1+2a | 0 | 1 | 0 | 1 | 0 | 1 | 1 | 1 | 1 | 1 | 1 | 0 | 1 |
| SG-M983 | Laos | 2015 | human | 1+2a | 0 | 1 | 0 | 1 | 0 | 1 | 1 | 1 | 1 | 1 | 0 | 0 | 1 |
| SG-M986 | Laos | 2015 | human | 1+2a | 0 | 1 | 0 | 1 | 0 | 1 | 1 | 1 | 1 | 1 | 0 | 0 | 1 |
| SG-M985 | Laos | 2015 | human | 1+2a | 0 | 1 | 0 | 1 | 0 | 1 | 1 | 1 | 1 | 1 | 1 | 0 | 1 |
| SG-M991 | Laos | 2015 | human | 1+2a | 0 | 1 | 0 | 1 | 0 | 1 | 1 | 1 | 1 | 1 | 1 | 0 | 1 |
| SG-M990 | Laos | 2015 | human | 1+2a | 0 | 1 | 0 | 1 | 0 | 1 | 1 | 1 | 1 | 1 | 1 | 0 | 1 |
| NA0068263 | Thailand | 2015 | human | 1+2a | 0 | 1 | 0 | 1 | 0 | 1 | 1 | 1 | 1 | 1 | 0 | 0 | 1 |
| SG-M613 | Singapore | 2015 | human | 1+2a | 0 | 1 | 0 | 1 | 0 | 1 | 1 | 1 | 1 | 1 | 1 | 0 | 1 |
| SG-M597 | Singapore | 2015 | human | 1+2a | 0 | 1 | 0 | 1 | 0 | 1 | 1 | 1 | 0 | 1 | 1 | 0 | 1 |
| SG-M621 | Singapore | 2015 | human | 1+2a | 0 | 1 | 0 | 1 | 0 | 1 | 1 | 1 | 0 | 1 | 1 | 0 | 1 |
| SG-M1011 | Vietnam | 2015 | human | 1+2a | 0 | 1 | 0 | 1 | 0 | 1 | 1 | 1 | 0 | 1 | 1 | 0 | 1 |
| SG-M1003 | Vietnam | 2015 | human | 1+2a | 0 | 1 | 0 | 1 | 0 | 1 | 1 | 1 | 0 | 1 | 1 | 0 | 1 |
| SG-M1012 | Vietnam | 2015 | human | 1+2a | 0 | 1 | 0 | 1 | 0 | 1 | 1 | 1 | 0 | 1 | 1 | 0 | 1 |
| SG-M1013 | Vietnam | 2015 | human | 1+2a | 0 | 1 | 0 | 1 | 0 | 1 | 1 | 1 | 0 | 1 | 1 | 0 | 1 |
| NA0064797 | Thailand | 2015 | human | 1+2a | 0 | 1 | 0 | 1 | 0 | 1 | 1 | 1 | 1 | 1 | 0 | 0 | 1 |
| SG-M633 | Singapore | 2015 | human | 1+2a | 0 | 1 | 0 | 1 | 0 | 1 | 1 | 1 | 1 | 1 | 1 | 0 | 1 |
| SG-M997 | Laos | 2015 | human | 1+2a | 0 | 1 | 0 | 1 | 0 | 1 | 1 | 1 | 1 | 1 | 0 | 0 | 1 |
| SG-M998 | Laos | 2015 | human | 1+2a | 0 | 1 | 0 | 1 | 0 | 1 | 1 | 1 | 1 | 1 | 0 | 0 | 1 |
| SG-M994 | Laos | 2015 | human | 1+2a | 0 | 1 | 0 | 1 | 0 | 1 | 1 | 1 | 1 | 1 | 1 | 0 | 1 |
| SG-M995 | Laos | 2015 | human | 1+2a | 0 | 1 | 0 | 1 | 0 | 1 | 1 | 1 | 1 | 1 | 0 | 0 | 1 |
| SG-M999 | Laos | 2015 | human | 1+2a | 0 | 1 | 0 | 1 | 0 | 1 | 1 | 1 | 1 | 1 | 0 | 0 | 1 |
| NA0064272 | Thailand | 2015 | human | 1+2a | 0 | 1 | 0 | 1 | 0 | 1 | 1 | 1 | 1 | 1 | 0 | 0 | 1 |
| NA0054981 | Thailand | 2015 | human | 1+2a | 0 | 1 | 0 | 1 | 0 | 1 | 1 | 0 | 1 | 1 | 0 | 0 | 1 |
| NA0061863 | Thailand | 2015 | human | 1+2a | 0 | 1 | 0 | 1 | 0 | 1 | 1 | 1 | 1 | 1 | 0 | 0 | 0 |
| NA0058901 | Thailand | 2015 | human | 1+2a | 0 | 1 | 0 | 1 | 0 | 1 | 1 | 0 | 1 | 1 | 0 | 0 | 1 |
| NA0054832 | Thailand | 2015 | human | 1+2a | 0 | 1 | 0 | 1 | 0 | 1 | 1 | 0 | 1 | 1 | 0 | 0 | 1 |
| SA0012037 | Thailand | 2015 | human | 1+2a | 0 | 1 | 0 | 1 | 0 | 1 | 1 | 0 | 1 | 1 | 0 | 0 | 1 |
| SA0051004 | Thailand | 2015 | human | 1+2a | 0 | 1 | 0 | 1 | 0 | 1 | 1 | 1 | 1 | 1 | 0 | 0 | 1 |
| SA0050550 | Thailand | 2015 | human | 1+2a | 0 | 1 | 0 | 1 | 0 | 1 | 1 | 1 | 1 | 1 | 0 | 0 | 1 |
| SA0042161 | Thailand | 2015 | human | 1+2a | 0 | 1 | 0 | 1 | 0 | 1 | 1 | 1 | 1 | 1 | 0 | 0 | 1 |
| SA0007717 | Thailand | 2015 | human | 1+2a | 0 | 1 | 0 | 1 | 0 | 1 | 1 | 0 | 1 | 1 | 1 | 0 | 1 |
| SA0013062 | Thailand | 2015 | human | 1+2a | 0 | 1 | 0 | 1 | 0 | 1 | 1 | 0 | 1 | 1 | 0 | 0 | 1 |
| SA0034366 | Thailand | 2015 | human | 1+2a | 0 | 1 | 0 | 1 | 0 | 1 | 1 | 1 | 1 | 1 | 0 | 0 | 1 |
| NA0064453 | Singapore | 2015 | human | 1+2a | 0 | 1 | 0 | 1 | 0 | 1 | 1 | 1 | 1 | 1 | 0 | 0 | 1 |
| SA0031662 | Singapore | 2015 | human | 1+2a | 0 | 1 | 0 | 1 | 0 | 1 | 1 | 0 | 1 | 1 | 0 | 0 | 1 |
| SG-M835 | Singapore | 2015 | human | 1+2a | 0 | 1 | 0 | 1 | 0 | 1 | 1 | 1 | 0 | 1 | 1 | 0 | 1 |
| SG-M844 | Singapore | 2015 | human | 1+2a | 0 | 1 | 0 | 1 | 0 | 1 | 1 | 1 | 1 | 1 | 1 | 0 | 1 |
| SG-M821 | Singapore | 2015 | human | 1+2a | 0 | 1 | 0 | 1 | 0 | 1 | 1 | 1 | 0 | 1 | 1 | 0 | 1 |
| SG-M742 | Singapore | 2015 | human | 1+2a | 0 | 1 | 0 | 1 | 0 | 1 | 1 | 1 | 1 | 1 | 1 | 0 | 1 |
| SG-M744 | Singapore | 2015 | human | 1+2a | 0 | 1 | 0 | 1 | 0 | 1 | 1 | 1 | 1 | 1 | 1 | 0 | 1 |
| SG-M883 | Singapore | 2015 | human | 1+2a | 0 | 1 | 0 | 1 | 0 | 1 | 1 | 1 | 1 | 1 | 0 | 0 | 1 |
| SG-M654 | Singapore | 2015 | human | 1+2a | 0 | 1 | 0 | 1 | 0 | 1 | 1 | 1 | 1 | 1 | 1 | 0 | 1 |
| SG-M870 | Singapore | 2015 | human | 1+2a | 0 | 1 | 0 | 1 | 0 | 1 | 1 | 1 | 0 | 1 | 1 | 0 | 1 |
| SG-M917 | Singapore | 2015 | human | 1+2a | 0 | 1 | 0 | 1 | 0 | 1 | 1 | 1 | 0 | 1 | 1 | 0 | 1 |
| SG-M909 | Singapore | 2015 | human | 1+2a | 0 | 1 | 0 | 1 | 0 | 1 | 1 | 1 | 0 | 1 | 1 | 0 | 1 |
| SG-M900 | Laos | 2015 | human | 1+2a | 0 | 1 | 0 | 1 | 0 | 1 | 1 | 1 | 1 | 1 | 1 | 0 | 1 |
| SG-M908 | Laos | 2015 | human | 1+2a | 0 | 1 | 0 | 1 | 0 | 1 | 1 | 1 | 1 | 1 | 1 | 0 | 1 |
| SG-M920 | Laos | 2015 | human | 1+2a | 0 | 1 | 0 | 1 | 0 | 1 | 1 | 1 | 0 | 1 | 1 | 0 | 1 |
| SG-M961 | Laos | 2015 | human | 1+2a | 0 | 1 | 0 | 1 | 0 | 1 | 1 | 1 | 1 | 1 | 1 | 0 | 1 |
| SG-M979 | Laos | 2015 | human | 1+2a | 0 | 1 | 0 | 1 | 0 | 1 | 1 | 1 | 1 | 1 | 1 | 0 | 1 |
| SG-M980 | Laos | 2015 | human | 1+2a | 0 | 1 | 0 | 1 | 0 | 1 | 1 | 1 | 1 | 1 | 1 | 0 | 1 |
| SG-M964 | Laos | 2015 | human | 1+2a | 0 | 1 | 0 | 1 | 0 | 1 | 1 | 1 | 0 | 1 | 0 | 0 | 1 |
| SG-M971 | Laos | 2015 | human | 1+2a | 0 | 1 | 0 | 1 | 0 | 1 | 1 | 1 | 0 | 1 | 1 | 0 | 1 |
| SG-M976 | Laos | 2015 | human | 1+2a | 0 | 1 | 0 | 1 | 0 | 1 | 1 | 1 | 1 | 1 | 1 | 0 | 1 |
| SG-M978 | Laos | 2015 | human | 1+2a | 0 | 1 | 0 | 1 | 0 | 1 | 1 | 1 | 1 | 1 | 0 | 0 | 1 |
| SG-M973 | Laos | 2015 | human | 1+2a | 0 | 1 | 0 | 1 | 0 | 1 | 1 | 1 | 1 | 1 | 1 | 0 | 1 |
| SG-M967 | Laos | 2015 | human | 1+2a | 0 | 1 | 0 | 1 | 0 | 1 | 1 | 1 | 1 | 1 | 0 | 0 | 1 |
| SG-M587 | Laos | 2015 | human | 1+2a | 0 | 1 | 0 | 1 | 0 | 1 | 1 | 1 | 0 | 1 | 1 | 0 | 1 |
| SG-M970 | Laos | 2015 | human | 1+2a | 0 | 1 | 0 | 1 | 0 | 1 | 1 | 1 | 1 | 1 | 0 | 0 | 1 |
| 20166199 | USA | 2016 | other human | 1+2a | 0 | 1 | 0 | 1 | 0 | 1 | 1 | 1 | 1 | 1 | 1 | 0 | 1 |
| PHEGBS0054 | UK | Unknown | Unknown | 1+2a | 0 | 1 | 0 | 1 | 0 | 1 | 1 | 1 | 1 | 1 | 0 | 0 | 1 |
| NGBS128 | Canada | 2010 | human | 1+2b | 1 | 0 | 1 | 0 | 1 | 1 | 1 | 1 | 1 | 1 | 1 | 1 | 1 |
| ZJSX030 | China | 2017 | human | 1+2a | 0 | 1 | 0 | 1 | 0 | 1 | 1 | 1 | 1 | 1 | 0 | 0 | 1 |
